# Peer review of "Influence of Pore Networking and Electric Current Density on the Crack Pattern in Reinforced Concrete Test Due to Pressure Rust Layer at Early Ages of an Accelerated Corrosion Test"

_materials, 2019, doi:10.3390/ma12152477_

Round 1
Reviewer 1 Report
The manuscript is very interesting as it analyses the influence of the porous micro-structure and current density in the initiation stage of rebar corrosion process, and the crack formation induced by chloride penetration in concrete. It is worth of publication; however some revisions are required before that stage.
I suggest to use the term "program" instead of "campaign" throughout the manuscript.
I suggest to improve the introduction with earlier (than Su and Zhang 2015 [11]) studies on the thick-cylinder model. A state of art is provided for instance in Bossio et al. "Nondestructive assessment of corrosion of reinforcing bars through surface concrete cracks" (2017) Structural Concrete, 18 (1), pp. 104-117, potentially to add.
Line 148. Probably it is better "To enforce".
Line 165. Do you mean "imposed" (i.e. really applied?) instead of "assumed" (simply desired, not actual)?
How did you obtain thin slices of approximately 1 mm for SEM? It is important to clarify if there was not any pollution.
Can you please provide also the Coefficient of variation of the circumferential strains measured by the 4 embedded gauges in each specimen?
In figure 6 units of strains are missing, I suppose it is not up to 0.01, as it is a huge strain for a concrete in tension (1%??? it is probably not reached in a compressed confined concrete). There is no comment on strain values in the text.
Line 228, In my opinion the linear pattern can be found for 50 and 100 (after CAR filling), but the slope of the curve 100 is about doubled... However it is expected that time to fill CAR should be also halved. Furthermore it is not clear why peak strain was doubled as tensile strength (and strain) at cracking should be similar; however strain includes the post peak softening...
Please revise Table 7 (Tiempo?) and delete the two digits after dot in first column (you did not consider minutes).
Line 275. You multiply time by intensity, but what about the surface? Please provide explicitly the values of factors in the multiplication.
In figure 9 units of strains are missing (and the same comment for figure 6 repeats).
Line 320, please clarify what the (2 or 3) stages are?
Line 346. Please delete doubled "the radius"
Line 357. Apart f, you can also provide an expansion coefficient of steel into rust, as you have both volume of steel loss (thickness of the ring r0-r1) and volume of rust (thickness of the ring r2-r1, or something related to it, to consider CAR). Please provide also these values of volumes ratio.
Line 380. The slight fall in the strain measured at the moment of cracking is probably due to the reduction in stiffness of the thick cylinder under radial pressure by rust layer, that requires some more expansion before reaching again the radial pressure, hence the circumferential pressure correlated to radial one?
Line 385, please revise "tensile" into "theoretical tensile stress" as it is not an actual stress when it is higher than strength.
Table 12. Units are missing for "theoretical tensile stress".
Eq. (7) seems wrong, as you cannot read the strain of a crack, as the gauge was broken (in fact in equation 6 the strain is the strain read by strain gauge only when w=0). Conversely the strain in equation 9 should be a "total theoretical" strain as a "theoretical tensile stress" divided by Elastic Modulus.
Line 420. "analytical model based"??
Line 426. You cannot strictly state this because you did not a test with strain gauges on the external surface of concrete, however I agree that it is probably true, but should be clarified that it is supposed.
Author Response
The manuscript is very interesting as it analyses the influence of the porous micro-structure and current density in the initiation stage of rebar corrosion process, and the crack formation induced by chloride penetration in concrete. It is worth of publication; however some revisions are required before that stage.
The authors are very grateful to the reviewer for the detailed comments, which have helped them to address important details and to improve the quality of the paper. The manuscript has been revised and changes are highlighted in red throughout the text.
A technical revision has been conducted in order to complete and clarify the current study.
Moreover, the use of the language in the manuscript and technical remarks has been revised following the corrections suggested by the reviewer and the requested corrections have been included along the text.
I suggest to use the term "program" instead of "campaign" throughout the manuscript.
The manuscript has been revised following the reviewer’s suggestion.
I suggest to improve the introduction with earlier (than Su and Zhang 2015 [11]) studies on the thick-cylinder model. A state of art is provided for instance in Bossio et al. "Nondestructive assessment of corrosion of reinforcing bars through surface concrete cracks" (2017) Structural Concrete, 18 (1), pp. 104-117, potentially to add.
The authors are very grateful with the reviewer for this suggestion. The introduction has been modified by introducing a comment to earlier studies on the thick-cylinder model and including the reference [22]
Line 148. Probably it is better "To enforce".
Thank for this suggestion. The authors agree with the reviewer’s opinion and “To enforce” has been introduced.
Line 165. Do you mean "imposed" (i.e. really applied?) instead of "assumed" (simply desired, not actual)?
The authors agree with the reviewer’s opinion and “imposed” has been introduced instead of “assumed”.
How did you obtain thin slices of approximately 1 mm for SEM? It is important to clarify if there was not any pollution.
The authors are very grateful with the reviewer for this observation. Due to a mistake the thickness of the slices appeared in the text as 1mm instead of 10 mm (1 cm) (Line 174). This has been corrected.
The following figure shows a sketch of the cutting procedure. The text has been modified in order to clarify the cutting procedure
“First, prismatic samples containing the rebar with a square base of 50x50 mm2 were cut. Then, thin slices of approximately 10 mm thickness were obtained for SEM observation. All the cuts were made with the precision diamond cut-off machine STRUERS SECOTOM-10, with a 0.8 mm thick disc running across the prismatic sample. In order to avoid a washing of the corrosion products, liquid petroleum jelly was used to cool the cutting disc. Subsequently, these slices were prepared for SEM observation (see Figure 4b)
Figure 4b.Sketch of the cutting procedure of the specimens for SEM observation (dimensions in cm).
Can you please provide also the Coefficient of variation of the circumferential strains measured by the 4 embedded gauges in each specimen?
The coefficient of variation of the measured circumferential strain in each type of specimen was lower than 15%.
In figure 6 units of strains are missing, I suppose it is not up to 0.01, as it is a huge strain for a concrete in tension (1%??? it is probably not reached in a compressed confined concrete). There is no comment on strain values in the text.
Thank you for your comment. The units of circumferential strain have been included in Figures 6, 9, 10 and 11. In addition, the authors are in agreement with the value of 0.01 as limit for strain of concrete under tension.
Line 228, In my opinion the linear pattern can be found for 50 and 100 (after CAR filling), but the slope of the curve 100 is about doubled... However it is expected that time to fill CAR should be also halved. Furthermore it is not clear why peak strain was doubled as tensile strength (and strain) at cracking should be similar; however strain includes the post peak softening...
Thank you for your comment. Admittedly, it would be possible to adjust a linear pattern for 50 specimens with the slope of the curve 100 approximately halved. However in the author’s opinion the bilinear pattern has an especially good fit, with an initial horizontal part which represents a first stage during which the rust products fill the CAR, without causing stress. This may be better observed in Figure 9. In this work the specimens subjected to a current density of 100 μA/cm2show a linear pattern of the circumferential strain from the very beginning. Regarding the circumferential strain measured by the gauges, is important to take into consideration than might have an additional component due to micro-cracking before the formation of the macroscopic cracking, together with the possible injection of corrosion gel into the micro-cracks during the process. After the initiation of crack part of the circumferential stress is released. A comment has been introduced in the text.
Please revise Table 7 (Tiempo?) and delete the two digits after dot in first column (you did not consider minutes).
Thank you for the comment, this table has been corrected.
Line 275. You multiply time by intensity, but what about the surface? Please provide explicitly the values of factors in the multiplication.
Thank you for mentioning this, the sentence is incomplete and can lead to confusion. The current density is multiplied by the surface of the rebar subjected to corrosion, that is the same in all cases and equal to 37,7 cm2, to obtain the total current intensity, as the following table shows:
Surface rebar | Current density | Total current/circuit |
37.7 | 100 | 3.77 |
37.7 | 50 | 1.89 |
Then the total electric charge applied is obtained by multiplying the time by intensity.
The authors have corrected the text in order to clarify:
“This could be achieved by multiplying the time by intensity to obtain the total electric charge. The intensity is obtained by multiplying the current density by the surface of the rebar subjected to corrosion, that is the same in all cases and equal to 37.7 cm2(see Figure 2).”
In figure 9 units of strains are missing (and the same comment for figure 6 repeats).
Thank you for your observation, due to a mistake some units of strains were missing in some figures, that have been corrected in order to include them.
Line 320, please clarify what the (2 or 3) stages are?
The authors appreciate the comment and have modified the text in order to clarify. The new paragraph is the following:
“As showed in Figure 1 three stages can be distinguished in the corrosion process. The second stage is characterised by stress initiation when the corrosion accommodation region is completely filled with rust products that start to exert stress. Subsequently, a third stage can be identified by the formation of cracks when stress reaches the tensile strength of the concrete and rust fills the cracks as they are created. Recently, some authors have proposed that the three-stage model might be modified into a two-stage model [27]. In line with such an approach, the penetration of rust products into the porous microstructure and formation of a corrosion layer at the interface might occur simultaneously once the steel corrosion has been initiated. Based on such rationale, in this work the CAR and CL have been assessed jointly.”
Line 346. Please delete doubled "the radius"
Thank you, this has been corrected
Line 357. Apart f, you can also provide an expansion coefficient of steel into rust, as you have both volume of steel loss (thickness of the ring r0-r1) and volume of rust (thickness of the ring r2-r1, or something related to it, to consider CAR). Please provide also these values of volumes ratio.
Thank for this comment. The authors think that the requested values might of interest, but since the rust products are inside of the porous network, it is quite difficult to obtain a precise value of the rust ratio. The authors think that a precise assessment of the rust volume ratio, by measuring the material inside of the pore network and taking into account the stress confinement of the concrete influence, is out of the scope of the paper.
Line 380. The slight fall in the strain measured at the moment of cracking is probably due to the reduction in stiffness of the thick cylinder under radial pressure by rust layer, that requires some more expansion before reaching again the radial pressure, hence the circumferential pressure correlated to radial one?
Thank you for this comment. According with the reviewer suggestion, the authors have corrected the sentence to clarify the probably reason of the slight fall observed in the strain, following text within quotas:
“Additionally, a slight fall in the strain measured by the unbroken strain gauges is observed at the moment of cracking (see Figure 10). This slight fall in the strain measured is produced at the moment of cracking probably due to a reduction in stiffness of the thick cylinder under radial pressure by the rust layer. Part of the circumferential stress is released after the initiation of crack, and some more expansion is required before reaching again the radial pressure.”
The analytical model proposed in this work provided an estimate of the mean value of the crack width, which might be accurate enough for modeling. The real width may be different for each crack.
Line 385, please revise "tensile" into "theoretical tensile stress" as it is not an actual stress when it is higher than strength.
Thank you for your comment. This has been modified: “crack width will appear when the theoretical tensile stress (s*) exceeds this value.”
Table 12. Units are missing for "theoretical tensile stress".
Thank you for the observation. Table 10 has been modified in order to introduce the units for the theoretical tensile stress
Eq. (7) seems wrong, as you cannot read the strain of a crack, as the gauge was broken (in fact in equation 6 the strain is the strain read by strain gauge only when w=0). Conversely the strain in equation 9 should be a "total theoretical" strain as a "theoretical tensile stress" divided by Elastic Modulus.MPa
Thank you for your comment. The authors agree with the correction and Equation 7 has been corrected, changing “epsilon_sg” by “epsilon”.
Line 420. "analytical model based"??
Thank you, the sentence is incomplete. The text has been modified: “An analytical model based on the thick-walled cylinder approach”
Line 426. You cannot strictly state this because you did not a test with strain gauges on the external surface of concrete, however I agree that it is probably true, but should be clarified that it is supposed.
The authors agree with the reviewer’s opinion, and this sentence has been modified in order to clarify that it is supposed. The new wording is the following:
“The results obtained showed that embedding the strain gauges in the concrete, placed as closely as possible to the steel rebar, might provide more detailed data of the circumferential strain in the concrete at local level than use of strain gauges on the concrete surface.”
Reviewer 2 Report
This is an interesting paper concerning the discussions of the influence of the current density on the initiation of crack formation and the model for predicting the crack formation referencing the data from SEM observations, EDS analysis and the strain gauge measurements. The paper has its scientific novelty and contributions on the knowledge of crack formation for accelerated corrosion test.
In order to improve the quality of the presented paper, the author may consider my following suggestions:
1) Line 26, the space after “pores” should be deleted.
2) Line 37, the space after “frequent” should be deleted.
3) Line 145, the results of the compressive strength, elasticity modulus and tensile strength of SFC are all less than CC, which is unexpected when 10% of silica fume was added. Author should have a discussion on it. In addition, there is no information about the silica fume e.g. the percentage of silica dioxide to be mentioned in the section of material. Author should provide this information as well.
4) Line 196, author mentioned the concrete as high-strength concrete which the term did not appear in the previous content. Referring the 28-day compressive strength is lower than 70MPa, the concrete may not be classified to high-strength concrete. It is suggested to change high-strength concrete to the concrete.
5) Line 246, what does BSE stand for? Author should give the full name of the abbreviation if it first appears in the paper.
Author Response
This is an interesting paper concerning the discussions of the influence of the current density on the initiation of crack formation and the model for predicting the crack formation referencing the data from SEM observations, EDS analysis and the strain gauge measurements. The paper has its scientific novelty and contributions on the knowledge of crack formation for accelerated corrosion test.
The authors are very grateful to the reviewers for the detailed comments, which have helped them to address important details and to improve the quality of the paper. The manuscript has been revised and changes are highlighted in red throughout the text.
A technical revision has been conducted in order to complete and clarify the current study.
Moreover, the use of the language in the manuscript and technical remarks has been revised following the corrections suggested by the reviewer and the requested corrections have been included along the text.
In order to improve the quality of the presented paper, the author may consider my following suggestions:
The authors appreciate these comments. They have all been revised and modified
1) Line 26, the space after “pores” should be deleted. Corrected
2) Line 37, the space after “frequent” should be deleted. Corrected
3) Line 145, the results of the compressive strength, elasticity modulus and tensile strength of SFC are all less than CC, which is unexpected when 10% of silica fume was added. Author should have a discussion on it. In addition, there is no information about the silica fume e.g. the percentage of silica dioxide to be mentioned in the section of material. Author should provide this information as well.
Thank you for your comment. In this work two concrete mixtures were performed in laboratory conditions trying to approximately maintain the same properties, with approximately the same percentage of total porosity but with different pore microstructure. So, the purpose was not to obtain a concrete with higher properties and a lower total porosity. In this work were elaborated a conventional concrete (CC) with Portland cement CEM I 52.5 R (in accordance with the standard EN 197-1:2011), and a silica fume concrete (SFC) with Portland cement CEM I 42.5 R (in accordance with the standard EN 197-1:2011) and silica fume (SF) as mineral admixture (10% of cement weight and two as efficiency factor). Is important to keep in mind that SF is used as replacement of part of the cement. The rest of the mix proportioning was remained constant. As a result, two concretes with quite similar overall porosity, but different pore size distribution were obtained. The results of the Mercury Intrusion Porosimetry Test for both concretes have been included in the manuscript (Figure 5). In this figure is remarkable a considerable higher amount of small pores in SFC, together with a lower amount of big pore and macropore in comparison with CC. This means that SFC has a denser network, with smaller average diameter of pore (Table 6), which might influence on the penetration of rust products in the microstructure. A greater quantity of small pores might make the penetration of the corrosion products more difficult in this case. As a consequence of this, the corrosion-accommodating region at the interface is consumed more rapidly and so, corrosion products caused circumferential deformation and exerted a pressure on the surrounding concrete from an early age.
The chemical composition of the silica fume is shown below:
Silica Fume (%) | |
CaO | 0,51 |
Fe2O3 | 5,27 |
K2O | 0,60 |
MgO | 0,73 |
Na2O | 0,37 |
SiO2 | 91,0 |
TiO2 | 0,043 |
Al2O3 | 0,777 |
MnO | 0,17 |
ZnO | -- |
SO3 | -- |
The authors have modified the wording in order to clarify this point and to include the percentage of silica dioxide.
“Two concrete mixtures were designed trying to obtain similar properties and approximately the same percentage of porosity though with a different pore microstructure. As a result, two concretes with quite similar overall porosity, but different pore size distribution were obtained. Portland cement CEM I 52.5 R and Portland cement CEM I 42.5 R, in accordance with the standard EN 197-1:2011, were used for the preparation of the two concrete mixtures. The first cement type was used to elaborate a conventional concrete (CC) without admixtures and the second one to elaborate a silica fume concrete mixture (SFC) by using SF, with 91% of silica dioxide in its composition, as mineral admixture.”
4) Line 196, author mentioned the concrete as high-strength concrete which the term did not appear in the previous content. Referring the 28-day compressive strength is lower than 70MPa, the concrete may not be classified to high-strength concrete. It is suggested to change high-strength concrete to the concrete.
The authors appreciate this comment and agree with the reviewer’s opinion. This has been modified.
5) Line 246, what does BSE stand for? Author should give the full name of the abbreviation if it first appears in the paper.
BSE is defined in the second paragraph or section 2.4: “A field-emission scanning electron microscope (JEOL Superprobe JXA-8900 M) and operated in backscattered electron (BSE) mode was used to create the element maps of the steel/concrete interface at different ages.”
Reviewer 3 Report
(1) There are some references to discuss the corrosion rate effect on the cracking in view of the time-dependency of concrete and fracture energy. In the experiment, how coould the authors decide the rate (days scale) of corrosion of the experiments in tertms of creep or time-dependency of concrete? The authors’ stateement is meaningful when the readers consider how to link the expecimental facts to the real time scale of years.
(2) In the experiment, is there any possiblity of the corrosion gel injected into the micro-cracks produced by the self-equilibrated stress? Some commnets are recommended.
Author Response
The authors would like to thank the reviewer for your comments, which have helped them to address important details and to improve the quality of the paper. The manuscript has been revised following the reviewer’s suggestions and changes are highlighted in red throughout the text.
(1) There are some references to discuss the corrosion rate effect on the cracking in view of the time-dependency of concrete and fracture energy. In the experiment, how coould the authors decide the rate (days scale) of corrosion of the experiments in tertms of creep or time-dependency of concrete? The authors’ stateement is meaningful when the readers consider how to link the expecimental facts to the real time scale of years.
Thank you for your comment. In the tests carried out in this work have been used two different corrosion current densities usually used for accelerated corrosion studies. The text has been modified to justify the selection:
“The experimental program used accelerated corrosion tests with current densities of 50μA/cm2 and 100μA/cm2, usually used for accelerated corrosion studies [4, 13, 16-18] on two concretes mixtures: one without admixtures and another with silica fume (SF).“
Results are obtained under accelerated corrosion tests with these terms validity, as cannot be directly transferred to a realistic case. The authors are planning further studies to extend the present in order to improve understanding in this research line.
(2) In the experiment, is there any possiblity of the corrosion gel injected into the micro-cracks produced by the self-equilibrated stress? Some commnets are recommended.
The authors would like to thank the reviewer once again. In the experimental tests is likely to occur micro-cracking before the formation of the macroscopic cracking, together with the possible injection of corrosion gel into the micro-cracks during the process. The strains measured by the gauges might be increased due to this phenomenon. After the initiation of crack part of the circumferential stress is released.
The following text within quotas has been introduced in the manuscript:
“During the formation of cracks, it is worth to take into consideration than the circumferential strain measured by the gauges might have an additional component due to micro-cracking before the formation of the macroscopic cracking. This process is likely to occur together with a possible injection of corrosion gel into the micro-cracks during the process. After the initiation of crack part of the circumferential stress is released”
Reviewer 4 Report
Major comments
There are many long sentences in the manuscript that makes the reader to read several times in order to understand them. For example, lines 12-14 and lines 68-70. Keep in mind, the sentences do not necessary to be wrong. This is my opinion as a reader/reviewer. In addition, there are many up and down between the concepts. In other words, the authors need to simply explain the results. For example, the authors mentioned some information about methodology (lines 14-17). Then, they mentioned the objective of the work (lines 17-18). After that, they went back to methodology again (lines 18-25). Finally, they discussed some results and mixed with methodology again. Again, this kind of writing style is not wrong. However, it does not help the reader to easily get the information. The two mentioned issues can be seen in entire sections of the manuscript.
Generally, most of the cited references are published at years 1983-2011. The authors need an extensive and recent literature review to show and prove novelty of this work. Now days, the researchers combine general description, research significant and novelty of work in introduction section. In this work, the authors only are only focused on the general ideas concerning corrosion.
Specific comments
Identify y-axis, namely “Ơ” and fct in Figure 1.
Table 1. subscript number ‘’2’’ in CaCl2 .
Line 107 is not clear (*Efficiency factor of silica fume is taken 2).
Line 155. subscript number ‘’2’’ in CaCl2.
Table 7. Translate “Tiempo”.
Table 7. It may be easier if convert the period from hour to day.
Line 290-293. Which results? Do you mean the results of Figure 10?
Lines 312-317 are not complete. It is not clear which model is selected to be used in your study?
Line 357. The “r*” is not clear.
Author Response
There are many long sentences in the manuscript that makes the reader to read several times in order to understand them. For example, lines 12-14 and lines 68-70. Keep in mind, the sentences do not necessary to be wrong. This is my opinion as a reader/reviewer. In addition, there are many up and down between the concepts. In other words, the authors need to simply explain the results. For example, the authors mentioned some information about methodology (lines 14-17). Then, they mentioned the objective of the work (lines 17-18). After that, they went back to methodology again (lines 18-25). Finally, they discussed some results and mixed with methodology again. Again, this kind of writing style is not wrong. However, it does not help the reader to easily get the information. The two mentioned issues can be seen in entire sections of the manuscript.
The authors would like to thank the reviewer for your detailed comments, which have helped them to address important details and to improve the quality of the paper. The manuscript has been revised trying to follow the reviewer’s suggestions and changes are highlighted in red throughout the text.
Generally, most of the cited references are published at years 1983-2011. The authors need an extensive and recent literature review to show and prove novelty of this work. Now days, the researchers combine general description, research significant and novelty of work in introduction section. In this work, the authors only are only focused on the general ideas concerning corrosion.
The authors understand the comment and believe that the wording has been significantly improved with the modifications made.
Thank you for the comment. References have been improved and updated through the introduction of four recent papers, cited in the introduction section:
- Andrade, Carmen, et al. "Estimating corrosion attack in reinforced concrete by means of crack opening." Structural Concrete 17.4 (2016): 533-540.
- Michele Win Tai Mak, Pieter Desnerck and Janet M. Lees, Corrosion-induced cracking and bond strength in reinforced concrete, Construction and Building Materials, 10.1016/j.conbuildmat.2019.02.151, 208, (228-241), (2019).
- Zhang, Weiping, Junyu Chen, and Xujiang Luo. "Effects of impressed current density on corrosion induced cracking of concrete cover." Construction and Building Materials 204 (2019): 213-223.
- Bossio, A., Lignola, G. P., Fabbrocino, F., Monetta, T., Prota, A., Bellucci, F., Manfredi, G. Nondestructive assessment of corrosion of reinforcing bars through surface concrete cracks. Structural Concrete, 2017, 18(1), pp. 104-117.
Specific comments
The authors appreciate these comments. They all have been revised and modified.
Identify y-axis, namely “Ơ” and fct in Figure 1.
The variable of the axis is “sigma” not “O”. The quality of the Figure 1 has been improved.
Table 1. subscript number ‘’2’’ in CaCl2 .Corrected
Line 107 is not clear (*Efficiency factor of silica fume is taken 2).
This efficiency factor is commonly considered between 1 and 3, and the most frequent value used is 2.A new reference has been introduced in order to justify it:
Babu, K. Ganesh, and PV Surya Prakash. "Efficiency of silica fume in concrete." Cement and concrete research 25.6 (1995): 1273-1283.
Line 155. subscript number ‘’2’’ in CaCl2.Corrected
Table 7. Translate “Tiempo”. Corrected
Table 7. It may be easier if convert the period from hour to day. Corrected in the table and in the text,
Line 290-293. Which results? Do you mean the results of Figure 10? Thank you for the comment. Results showed in Figures 9 and 10.
Lines 312-317 are not complete. It is not clear which model is selected to be used in your study? Eq. 1 is deduced from the following reference:
J.A. Den Uijl, A.J. Bigaj, A bond model for ribbed bars based on concrete confinement. HERON-ENGLISH EDITION-, 41, pp. 201–226.
Line 357. The “r*” is not clear.
This value is explained in a previous work of the authors. The reference is already included in the original manuscript, and now is included in this section in order to clarify. The text is modified as follows: where r* isthe radius of the strain gauge measurement (sum of the radius of the rebar with the half of the width of the strain gauge [Bazant et al., 2018])
Round 2
Reviewer 1 Report
Authors substantially solved my concerns
Reviewer 4 Report
the paper can be recommended for publication.